# Systematic Review of Sarcopenia Biomarkers in Hip Fracture Patients as a Potential Tool in Clinical Evaluation

**DOI:** 10.3390/ijms252413433

**Published:** 2024-12-15

**Authors:** Filip Brzeszczyński, David Hamilton, Oktawiusz Bończak, Joanna Brzeszczyńska

**Affiliations:** 1 Department of Trauma, Orthopaedics and Musculoskeletal Oncology, Copernicus Memorial Hospital, Pabianicka 62, 93-513 Łódź, Polandbonczako@gmail.com (O.B.); 2 Research Centre for Health, Glasgow Caledonian University, Govan Mbeki Building, Cowcaddens Road, Glasgow G4 0BA, UK; 3 Department of General Biochemistry, University of Łódź, ul. Narutowicza 68, 90-136 Łódź, Poland

**Keywords:** sarcopenia, biomarkers, hip fractures, muscle decline, orthopedic surgery

## Abstract

Hip fractures are associated with high morbidity and mortality. Sarcopenia is a significant factor contributing to poor prognosis; however, the clinical diagnosis of sarcopenia remains difficult in surgical patients. This systematic review aims to identify the biomarkers of sarcopenia as diagnostic and predictive tools in patients admitted for hip fracture surgery. A systematic search was conducted in the MEDLINE, EMBASE, and Google Scholar databases according to the PRISMA guidelines. Biomarker study quality was assessed using the BIOCROSS score. A total of 7 studies met the inclusion criteria and 515 patients were included, of whom 402 (78%) were female and 113 (22%) were male. The mean age of the participants was 83.1 years (SD: 5.9). Skeletal muscle biopsies were used for biomarker assessment in 14% (1/7) of studies and venous blood samples were used in the remaining 86% (6/7). The highlighted sarcopenia biomarkers included the low expression of insulin-like growth factor (IGF-I) and tumor necrosis factor-α (TNF-α), along with high serum myostatin and low serum vitamin D levels. Overall, the BIOCROSS score was satisfactory, with all studies obtaining at least a score of 13/20. The orthopedic literature is limited; however, the highlighted biomarkers in this review could be used as adjuncts in the diagnosis of sarcopenia in surgical patients.

## 1. Introduction

Hip fractures are associated with a high risk of death and have an estimated global incidence of 14.2 million, which is expected to increase along with the aging population [1]. Patients with hip fractures are some of the most difficult orthopedic and surgical patients to manage in terms of post-surgical treatment, due to multiple comorbidities and loss of independence after surgery. The 1-year mortality rate following hip fracture is reported to be as high as 58% [2]. Both sarcopenia and hip fractures are intricately linked to frailty syndrome. When these conditions coexist, sarcopenic patients with hip fractures are reported to have not only increased mortality rates but also an increased risk of hospital infections, cardiovascular events, and decreased functional outcomes following surgery and, overall, represent an increased burden on healthcare resources [3].

Sarcopenia is characterized by the loss of skeletal muscle mass, strength, and function. It is a common condition, with an estimated prevalence of 40% in community-dwelling adults [4]. Sarcopenia is associated with variable risk factors. Among them, a lack of exercise is considered to be the major risk factor, as evidenced by the early decline in muscle fiber and strength, starting around the age of 50, reported in sedentary individuals [5]. Alterations in hormonal levels, including growth, sex, and thyroid hormones, and in insulin-like growth factor result in progressive muscle decline. Moreover, a combination of pro-inflammatory cytokines, such as tumor necrosis factor-alpha (TNF-α) and interleukin-6 (IL-6), can lead to muscle atrophy [6]. Despite this poor prognosis, therapeutic methods are limited. Currently, no pharmacological products have been approved by the Food and Drug Administration for the treatment of sarcopenia, and resistance training has been demonstrated as the main non-pharmacological sarcopenia management method, with significant positive evidence [6,7]. Similarly, exercise is the primary prevention method for delaying the onset of sarcopenia [7].

The most recent consensus definition provided by the European Working Group on Sarcopenia in Older People 2 (EWGSOP2) states that in order to diagnose sarcopenia, both low muscle strength and muscle mass or muscle mass of poor quality have to be present. Low muscle strength can be measured by a hand-held dynamometer and low muscle mass or quality are measured through either bioelectrical impedance analysis, dual-energy X-ray absorptiometry, or abdominal computer tomography imaging [8]. The presence of sarcopenia is confirmed by low muscle function, usually identified by testing the patient’s walking speed. Diagnosing sarcopenia in surgical hip fracture patients using the EWGSOP2 guidelines is, in practice, very difficult. Muscle strength measurements after sustained trauma are often inaccurate and gait speed tests are not possible, due to the nature of the injury. Previous reviews have shown that patients with acute trauma requiring surgery rarely have the appropriate radiological scans performed to measure muscle mass and muscle quality parameters [3]. Knowledge of the presence of sarcopenia can facilitate patient risk stratification and help with decisions as to future targeted clinical interventions, as well as in surgical decision-making. Therefore, biomarkers that can identify sarcopenic patients and reflect the severity of sarcopenia in hip fracture patients would be a hugely useful alternative diagnostic approach.

Despite the growing recognition of sarcopenia as a significant health concern, there remains a lack of consensus regarding reliable biomarkers for its assessment. Various biochemical, imaging, and functional measures have been proposed as potential biomarkers of sarcopenia, but their utility and validity for hip fracture patients are not well-established. Suggested potential sarcopenia biomarkers are myostatin (MSTN), insulin-like growth factor 1 (IGF-I), C-terminal agrin fragment (CAF), interleukin-6 (IL-6), tumor necrosis factor alpha (TNF-α), and vitamin D [9,10,11]. Though there are some suggested sarcopenia biomarkers, the presence of acute trauma such as a hip fracture may affect the identification of positive sarcopenia cases [12]. Furthermore, the heterogeneous phenotypic presentation of sarcopenia, an insufficient understanding of its pathophysiology, and the overlap of other age-related conditions have restricted sarcopenia studies. Additionally, most of the studies were conducted on animals, which is why there are no clinically validated prognostic biomarkers for sarcopenia [13]. There is a notable gap in the literature regarding the use of biomarkers as an alternative tool for diagnosing sarcopenia in these patients, particularly as traditional clinical assessments are not feasible or valid. This review seeks to fill this gap by evaluating existing research on the biomarkers of sarcopenia in hip fracture patients. By identifying reliable biomarkers that could serve as diagnostic or prognostic tools, we aim to provide evidence that could improve the early detection of sarcopenia and personalized treatment approaches for this vulnerable population.

## 2. Materials and Methods

### 2.1. Search Terms and Inclusion Criteria

A systematic review was conducted following the updated preferred reporting items for systematic reviews (PRISMA) guidelines [14]. The search was performed on Ovid MEDLINE and EMBASE to identify English-language peer-reviewed papers up to 1 December 2024. Conducted by a single author (F.B.), the search utilized the terms “Sarcopenia” OR “muscle decline” OR “muscle atrophy” OR “myopenia” AND “hip fracture” OR “intertrochanteric fracture” OR “neck of femur fracture” OR “subtrochanteric fracture” AND “biomarker” OR molecular marker” OR biological signature” OR “metabolite marker” OR proteomic marker” OR “genomic marker”. No search filters or hedges were applied. Two authors (F.B. and J.B.) independently screened the titles and abstracts. Discrepancies in study selection and data extraction were resolved through discussion and consensus between the review team (F.B. and J.B.). In cases where a consensus could not be reached, a third reviewer was consulted to make a final decision (O.B.).

Studies investigating biomarkers in patients or subgroups of patients with study-specific sarcopenia diagnosis and a present acute hip fracture were included in the search. A hip fracture was defined as any fracture of the proximal femur, including neck-of-femur fractures and intertrochanteric fractures. A biomarker was defined as a characteristic that is objectively measured and evaluated as an indicator of normal biological processes, pathogenic processes, or pharmacologic responses to a therapeutic intervention [15]. The definitions of sarcopenia and the criteria for its assessment were considered according to EWGSOP2 guidelines [8]. Studies were eligible if the included group of patients underwent surgical treatment of a hip fracture. Studies with hip fracture patients who did not undergo surgery were not included in the review. Non-clinical studies, animal studies, reviews, case reports, unpublished data, and conference reports were excluded. Studies lacking clear descriptions of sarcopenia or hip fracture diagnosis were also excluded. Non-English written articles were excluded.

### 2.2. Data Extraction

Data extraction was performed by the author F.B. Each article was analyzed and the authors, study publication year, study design, number of participants and their ethnic background, sarcopenia parameters measured, sarcopenia diagnostic criteria used, and perioperative outcomes were exported for further analysis. Definitions of sarcopenia and the criteria for assessment were considered, as per EWGSOP guidance. Microsoft Excel was used to calculate the mean age and male-to-female ratio.

### 2.3. Risk of Bias Assessment

All papers were assessed for methodological quality by two authors (F.B. and J.B.) using the quality appraisal tool for cross-sectional studies using biomarker data (BIOCROSS) [16]. BIOCROSS consists of 10 items covering 5 domains: ‘Study rationale’, ‘Design/Methods’, ‘Data analysis’, ‘Data interpretation’, and ‘Biomarker measurement’, aiming to assess different quality features of biomarker cross-sectional studies. Each item was scored, based on three issues to consider (IC): if all IC were discussed in a feasible way, a score of 2 was awarded; if one or two IC were discussed, 1 point was given; otherwise, a score of 0 points was awarded. The maximum possible score was 20 points. Based on the BIOCROSS results, each study was evaluated using a scoring system, which was quantified by the number of points awarded. The higher the score, the higher the methodological quality of the study.

## 3. Results

### 3.1. Search Results

**Figure 1:**
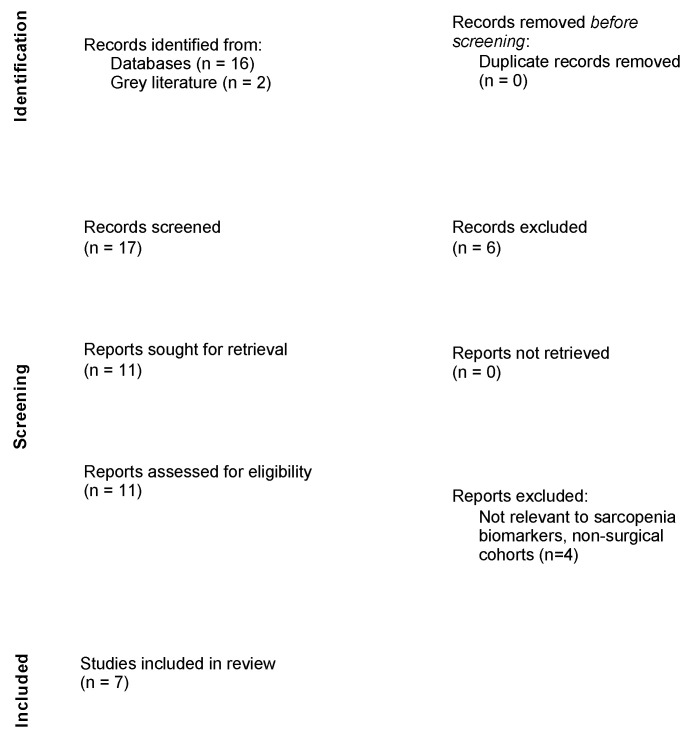
PRISMA flow diagram showing the systematic selection process of records.

The initial search yielded 17 publications. Two studies were subsequently added after searching the grey literature. After screening the article titles and abstracts, 11 studies were included for review (Figure 1) [14]. Following a full-text evaluation, a further four studies were excluded, leaving seven studies that met the inclusion criteria. The included papers comprised six comparative studies and one randomized control trial (Table 1). The European cohorts of patients comprised 72% (5/7) studies, Australian cohorts 14% (1/7), and Asian cohorts 15% (1/7). In total, there were 515 patients, of whom 402 (78%) were female and 113 (22%) were male. The mean age of the participants was 83.1 years (SD: 5.9). The average age and BMI of patients in sarcopenic and non-sarcopenic cohorts were reported in four studies; the average ages were 81.6 (SD: 6.8) years of age in sarcopenic patients and 78.8 (SD: 7.2) in non-sarcopenic patients. The mean reported BMI was 22.7 (SD:3.1) and 25.3 (SD: 1.5) in the sarcopenic and non-sarcopenic groups, respectively.

### 3.2. Sarcopenia Definitions

EWGSOP or the revised EWGSOP2 guidelines were used to define sarcopenic cohorts of patients in 86% (6/7) of studies [8,24]. The study by Yoo et al. in 2021 used the AWGS guidelines for sarcopenia definition, guidelines that were specific to their Asian cohort of patients [18,25]. Bermejo-Bescós et al. (2020), as well as Sánchez-Castellano et al. (2020), used the EWGSOP2 guidelines, along with Janssen’s and Masanés’ specific muscle mass cut-off points for American and Spanish sarcopenic cohorts [19,22,26,27]. The gold standard of three sarcopenia parameters (muscle strength, muscle quantity or quality, and muscle function) were measured in 14% (1/7) of studies; however, muscle function was measured postoperatively during patient rehabilitation. Two parameters were measured in the remaining 86% (6/7) of studies, whereby both low muscle mass and low muscle strength parameters had to be present to diagnose sarcopenia (Table 2). All seven studies estimated the patient’s muscle mass, for which 86% (6/7) of the studies used bioelectrical impedance analysis (BIA) and 14% (1/7) used dual-energy X-ray absorptiometry (DEXA) imaging. The cut-off values for low muscle mass were different in all studies (Table 2). Handgrip strength was measured in all studies. Low muscle strength was defined as <27 kg in men and <16 kg in women in five studies, as per EWGSOP2 guidelines [8]. In the remaining two studies, strength was defined as HGS < 30 kg in men and <20 kg in women as per EWGSOP1 guidelines, and <28 kg in men and <18 kg in women as per AWGS guidelines, in each respective article [24,25].

### 3.3. Sarcopenia Biomarkers

Only one study performed a biomarker analysis from muscle biopsies taken at the time of surgery. The remaining six studies obtained peripheral blood samples for analysis (Table 3). The time of blood sample collection was reported in five studies, with four studies reporting blood sample collection after hip fracture and within the perioperative period. Only de Sire et al. (2020) reported delayed blood-sample collection three months after surgery in their interventional randomized control trial [20]. The single study assessing muscle biopsy biomarkers showed reduced insulin-like growth factor-1 (IGF-I) expression in sarcopenic patients, leading to the decline of skeletal muscle neuromuscular junction [17]. Yee et al. (2020) also showed that women with hip fractures, compared to the controls, had lower serum IGF-I in a cohort exhibiting a 60% sarcopenia prevalence with hip fractures [21]. Similarly, Marzetti et al. (2014) assessed the peripheral blood serum CAF levels, a marker of neuromuscular junction degeneration. The results showed that CAF levels were significantly higher in sarcopenic patients relative to non-sarcopenic patients (172.2 ± 47.5 vs. 93.1 ± 44.0 ng/mL, *p* < 0.001) [23].

Pro-inflammatory biomarkers were assessed in 28% (2/7) of studies. Sanchez-Castellano et al. (2020) showed that TNF-α was lower in sarcopenic than in non-sarcopenic participants (7.9 ± 6.2 vs. 8.3 ± 5.8 pg/mL; *p* < 0.05), whereas the results of Bermejo-Bescós et al. (2020) highlighted that IL-6 baseline levels were significantly higher in the group of participants who died in the year after their hip fracture (*p* = 0.026); however, this was independent of patient sarcopenia status [19,22]. Elsewhere, de Sire et al. (2020) assessed peripheral blood myostatin levels as a potential biomarker to monitor sarcopenia in hip fracture patients undergoing rehabilitation. Significant reductions in serum myostatin levels were noted in sarcopenic patients receiving amino acid supplementation (*p* = 0.04) [20]. Only one study assessed bioavailable 25(OH)D levels, which were significantly decreased in the sarcopenia group compared with the non-sarcopenia group (*p* = 0.030) [18].

### 3.4. Quality Assessment

Based on the BIOCROSS quality assessment tool for cross-sectional studies using biomarker data, the average score was 13.6 (SD: 0.5) (Table 4). Overall, the BIOCROSS score was satisfactory for all included articles, with four studies ascertaining a score of 14, and the remaining three studies, a score of 13. All the studies discussed all ICs in the hypothesis objective, as well as providing an interpretation and evaluation of the results domain. The specimen characteristics and assay methods domains achieved the lowest scores, due to the descriptions of reproducibility assessments performed for evaluating biomarker stability being missing. Similarly, the laboratory measurement domain achieved a low score, due to information being missing regarding the blinding of laboratory staff during the biomarker analysis.

## 4. Discussion

This systematic review highlights potential sarcopenia diagnostic biomarkers in patients with hip fractures, making the detection of sarcopenia feasible in a cohort of patients with whom the traditional diagnostic process for sarcopenia is not possible. Previous reviews have highlighted that clinical sarcopenia diagnosis using traditional guidelines is difficult in the setting of emergency medicine, as well as generally in orthopedic surgery, as it is often not possible to directly measure muscle strength and function [3,28]. However, it is crucial to identify sarcopenic patients who are most at risk of perioperative complications, mortality and increased burden on healthcare system in order to improve patient outcomes. The studies in this review similarly note that the correlation of clinical parameters of sarcopenia to biomarker expression is challenging, with sarcopenia functional measurements only being performed after surgery in the rehabilitation period [20]. This is a general limitation of research in this area, as sarcopenia diagnosis would be most useful before surgical intervention to aid clinical decision-making when deciding whether total hip arthroplasty or hemiarthroplasty for displaced neck-of-femur hip fractures is offered [29]. Previous studies have examined prognostic biomarkers in hip fracture patients, such as low albumin levels, lymphocyte counts, and chemotactic cytokine CXCL-8 [30,31]. However, these studies did not address hip fractures in the context of sarcopenia or consider the impact of sarcopenia on poor postoperative outcomes.

The biomarkers that were selected relate to the pathogenesis of sarcopenia. This is linked to an alteration in the homeostasis between protein anabolism and catabolism in the muscle tissue, resulting in the progressive reduction of muscle mass [32]. This homeostasis is altered by several factors, including chronic exposure to inflammation, cortisol, and MSTN dysregulation, as well as over-expression of the ubiquitin–proteasome pathway (UPP), which promotes muscle tissue degradation [33].

MSTN is responsible for the reduction of skeletal muscle protein synthesis and growth, as well as for the inhibition of muscle tissue insulin sensitivity. MSTN induces the activation of Smad2 and Smad3 proteins, which are believed to inhibit the IGF-1/Akt/ mammalian target of rapamycin (mTOR) pathway, a critical pathway that is responsible for stimulating protein synthesis [34,35]. Its over-expression, furthermore, disrupts myogenesis by MYOD inhibition, affecting muscle satellite cell (MSC) proliferation and differentiation. MSTN is also associated with the negative regulation of AMPK activity and the reduction of GLUT4 translocation to the plasma membrane (reducing glucose uptake and insulin sensitivity) and can also reduce GLUT4 protein expression. This, in turn, reduces the availability of glucose transporters and contributes to skeletal muscle insulin resistance. The role of MSTN as a promising biomarker of sarcopenia in a cohort of 20 elderly patients with osteoporotic hip fractures was described by de Sire et al. (2020) [20]. Researchers reported that a two-month course of combined nutritional and rehabilitative intervention in patients over 70 years old with surgically treated hip fractures induced a reduction in serum myostatin levels, suggesting its potential role as a promising circulating biomarker to monitor age-related sarcopenia. The relationship between serum myostatin and postoperative multidisciplinary hip fracture rehabilitation and nutritional management is an understudied topic [36,37].

Muscle growth and repair is controlled by hormonal factors such as growth hormone (GH), IGF-I, estrogen, and testosterone [38]. IGF-I production plays a dominant role in muscle healing and maintenance. Preclinical experiments have shown that IGF-I is associated with muscle mass and strength development, the reduction of muscle degeneration, the prevention of tissue toxin-induced inflammatory expansion, and an increase in the proliferation capacity of MSCs [39]. Similarly, GH induces the process of differentiation in muscle cells to form postmitotic myotubules and myofibers. Moreover, the level of IGF-I, the local mediator of GH action, is observed to reduce significantly with age [40]. 

IGF-I and MSTN have contrasting roles in the regulation of skeletal muscle size and growth, in particular, elevated expressions of muscle mRNA and circulating concentrations of IGF-I were observed following MSTN inhibition [41]. Therefore, in order to maintain healthy muscle tissue, the level and interplay between IGF-1 and MSTN are crucial to regulating muscle mass by acting through various signaling pathways that regulate muscle growth and maintain muscle homeostasis.

Yee et al. (2020) analyzed a hormonal biomarker panel related to skeletal muscle regulation in older women with hip fractures and healthy controls [21]. The authors reported that sarcopenic hip fracture patients had significantly lower serum albumin, IGF-I, insulin-like growth factor binding protein 3 (IGFBP-3), and free testosterone levels, as well as impaired beta cell function according to homeostasis model assessment (HOMA beta). The suggested positive relationship observed between IGF-I and IGFBP-3 with skeletal muscle mass and function may suggest a stimulatory effect on muscle growth, thereby explaining their protective effect against muscle decline. The findings of lowered IGF-I and IGFBP-3 levels in the hip fracture group were similar to findings from previous studies in hip fracture cohorts, as well as in stroke patients [42,43]. A positive association between free testosterone levels and low muscle cross-sectional area on a mid-thigh CT and strength in hip fracture patients was also observed. Similarly, other studies also suggest a relationship between elevated free testosterone levels and lean body mass [44,45]. Yee et al. (2020) further reported decreased beta cell function, an indicator of intrinsic insulin production, in the hip fracture group [21]. Insulin prevents protein breakdown and increases protein synthesis through the activation of mTOR 1 signaling in skeletal muscle; thus, the study by Yee et al. (2020) further supports the role of insulin in the maintenance of skeletal muscle in the context of sarcopenic patients [21]. Generally, IGF-1 and IGFBP-3, HOMA beta cell function, and free testosterone levels may serve as potential biomarkers of sarcopenia in hip-fracture patients; however, further evaluation of the relationship between the IGF-I pathway, insulin homeostasis, and its effect on skeletal muscle regulation is needed.

Jarmusch et al. (2021) assessed the emerging role of IGF-I as a key growth factor in muscle metabolism and its neuroprotective effect on aged muscle fibers [17]. In recent years, the neuroprotective role of IGF has been described [46]. However, therapeutic trials in neurodegenerative and muscle atrophy-associated diseases and, more recently, in older adults have shown contradictory data as to any IGF-I effect on muscle strength [47,48]. Nevertheless, these reports, as well as data from the study by Jarmusch et al. (2021), suggest that sufficient IGF-I concentrations play a critical role at a molecular level in sarcopenia [17]. A correlation between reduced IGF-I serum concentrations and the markers of skeletal muscle denervation in sarcopenic patients, based on muscle biopsy and histological and electrophysiological assessment, was also reported. The results showed that men had a significantly lower motor unit number index (MUNIX) and higher motor unit size index (MUSIX). Patients with decreased IGF-I serum concentration were more affected by sarcopenia [17]. IGF-I activation of satellite cells is correlated with the induction of muscle growth via the stimulation of the Akt/mTOR pathway [49]. Thus, a significant correlation of reduced IGF-I concentrations in older adults and low Ki67 expression can be considered as an indicator of reduced muscle proliferation. However, whether IGF-I itself directly upregulates Ki67 is yet to be documented. Previous large cohorts as well as the small sample of geriatric hip fracture patients by Jarmusch et al. (2021) suggest that low levels of IGF-I are associated with sarcopenia [10,17]. The authors further showed that when denervation of aged muscle tissue occurs, the level of reinnervation, marked by increased NCAM expression, was correlated with increased IGF-I concentrations. This suggests an interplay between endocrine and neurological factors in the pathogenesis of sarcopenia. Furthermore, a significant correlation between reduced IGF-I concentrations and low MUNIX values and MUSIX values was found. The study showed that patients with reduced MUNIX values had significantly higher MUSIX values, representing the size of the motor units. Since these participants were more affected by sarcopenia, these findings point towards the presence of compensatory mechanisms in motoneurons, such as neuronal sprouting. Nevertheless, both MUNIX and MUSIX measurements have a potential role as a sarcopenia biomarker in correlation with reduced IGF-I serum concentration. In vivo muscle biopsy studies are needed to further investigate IGF-I’s pharmacological effect in the prevention and treatment of aging denervated muscle fibers.

Reduced reinnervation capacity due to age-related disruption at the neuromuscular junction (NMJ) is recognized as a significant factor of sarcopenia [50]. The C-terminal agrin fragment (CAF) is a key component of the neuromuscular junction (NMJ). Aging affects NMJ remodeling, resulting in CAF shedding into the circulation and the disruption of nerve–muscle cross-talk [51]. This results in a gradual buildup of denervated muscle fibers [51]. In light of this finding, CAF, as a biomarker of NMJ stability, has been suggested as an early diagnostic biomarker of sarcopenia, especially when it is significantly elevated in the serum of elderly sarcopenic individuals [52,53]. Marzetti et al. (2014) showed increased serum CAF levels in surgical sarcopenic patients with hip fractures [23]. Despite the relatively small sample size, these results suggest that serum CAF levels could potentially serve to identify a subset of hip fracture patients at high risk of adverse health outcomes.

Bermejo-Bescós et al. (2020) studied the relationship between plasma proteasome activity and glutathione, BuChE activity, and CAF levels, respectively, and mortality in older adults with hip fractures [19]. No association between the measured biomarkers and 1-year mortality was found. The only peripheral biomarker associated with mortality was IL-6; however, this was independent of patient sarcopenia status. However, the previous literature suggests a relationship between IL-6, TNF-α levels, and mortality [54]. In vivo studies suggest that *IL-6* and *TNFα* are important trophic factors initiating stable myogenic regeneration, ensuring tissue homeostasis maintenance and normal aging regarding muscle function [55,56,57]. In contrast, in vitro studies have shown that inflammation with increased *TNFα* expression can be regarded as a factor that is directly responsible for muscle degeneration and muscle death [57].

Sanchez-Castellano et al. (2020) investigated several neuromuscular, proinflammatory, and oxidative stress peripheral blood markers in 150 elderly hip fracture patients, assessing the differences between sarcopenic and non-sarcopenic participants [22]. Only peripheral TNF-α levels and catalase activity were noted to be significantly lower in sarcopenic patients. The authors suggested that these two markers may indicate an early inflammatory reaction that is hampered in sarcopenic patients. In a previous in vitro study of muscle biopsies, no significant activity of *TNFα* and *IL-6* was detected in elderly sarcopenic patients when compared with non-sarcopenic participants [57]. Nevertheless, the presence of chronic inflammatory processes that are associated with muscle aging was identified before a clinical diagnosis of sarcopenia was given [57]. It is plausible that, at the time when atrophy is diagnosed, the muscle tissue that has been changed by the numerous processes preceding this state becomes more fibrotic. This suppresses *TNFα* and *IL-6* detection in muscle biopsies, which can also hamper their release into the circulation.

This review has various limitations. The limited number of studies included in this systematic review resulted in each study evaluating a distinct biomarker, making it impossible to directly compare findings across studies investigating the same biomarker. However, this reflects a broader challenge in sarcopenia research, as most studies have been conducted on animals, leading to a lack of molecular studies using human models [13]. In the included studies, sarcopenia was often assessed in the postoperative period, which may not entirely reflect the patient’s sarcopenia status at the time of injury. Many of the studies included small sample sizes, which limits the generalizability of the findings. In addition, differences in the demographics of the study populations, such as mismatched male-to-female ratios and varied population ethnicities, could have led to population bias. Most of the sarcopenia biomarkers found in this population were derived from venous blood samples, as opposed to the more commonly obtained muscle biopsies. This may be because of the logistical difficulties associated with obtaining human samples in trauma cases. From a clinical perspective, venous blood sample-based biomarkers may be very useful, as these are more easily obtainable in routine hospital workflows and taking them would be less laborious, compared to muscle biopsies that are either obtained preoperatively or during the surgery itself. Biomarkers were also analyzed at various time points in the perioperative period. This introduced a temporal bias, whereby assessing the biomarkers at different stages of the post-fracture process may have led to varying interpretations of their diagnostic and prognostic utility. Also, there was an inconsistency in biomarker stability. Such a bias can affect research outcomes by introducing inconsistencies in data interpretation. Biomarker measurements taken at different time points can lead to variations in their recorded levels, potentially hindering the accurate assessment of their true diagnostic and prognostic value. This variability highlights the importance of standardizing the timing of biomarker collection, to ensure reliable and comparable results across studies. Some biomarkers may not be stable over time and their levels could decrease or fluctuate, based on when the sample is collected relative to the fracture event or trauma associated with the hip fracture’s surgical intervention. This issue complicates the ability to compare findings across studies. The overall quality of the included studies was satisfactory; however, frequently, studies were missing a description of the reproducibility assessments for evaluating biomarker stability, which may influence biomarker efficacy in clinical settings. Similarly, future biomarker studies should also take into consideration the biomarker’s predictive validity and how this would translate into a clinical setting.

## 5. Conclusions

The presence of sarcopenia in those who sustain a hip fracture event is associated with poor morbidity and mortality outcomes. The detection of sarcopenia using traditional assessment guidelines is not feasible in this clinical population. Our review suggests that several potential sarcopenia biomarkers exist that could help diagnose its presence; however, current research is limited and insufficient to support their widespread use in clinical practice. The highlighted sarcopenia biomarkers in this study included the low expression of IGF-I and TNF-α, high serum MSTN, and low serum vitamin D levels; however, the current literature is limited, and further research is required. This work offers sarcopenia biomarkers in hip fractures, which should be further investigated in future studies, focusing on standardizing biomarker assessment methods, particularly regarding the timing of blood sample collection, to improve comparability. Longitudinal research tracking biomarkers over time could help to identify early prognostic markers and improve patient management. Additionally, integrating clinical and molecular data may enhance diagnostic accuracy and facilitate personalized treatment strategies for sarcopenia.

## Figures and Tables

**Table 1:** Descriptive summary of the included studies.

Author Country	Study Design	Mean Patient Age (Years)	Study Size (Female:Male Ratio)	Biopsy Site and Timing of Sample Collection
Jarmusch et al., 2021 [17] Germany	Observational	80.6	31 20:11	Vastus lateralis muscle during surgery
Yoo et al., 2021 [18] Republic of Korea	Comparative	72.0	83 58:25	Peripheral blood sample -
Bermejo-Bescós et al., 2020 [19] Spain	Comparative	87.6	150 118:32	Peripheral blood sample 24 h before surgery
de Sire et al., 2020 [20] Italy	Randomised Control Trial	75.9	20 17:3	Peripheral blood sample 3 months after surgery
Yee et al., 2020 [21] Australia	Comparative	77.82	39 39:0	Peripheral blood sample on days 1–5 after surgery
Sánchez-Castellano et al., 2020 [22] Spain	Comparative	87.6	150 118:32	Peripheral blood sample on day 1 of hospital admission
Marzetti et al., 2014 [23] Italy	Comparative	83.7	42 32:10	Peripheral blood sample on day 1 of hospital admission

**Table 2:** Summary of the parameters and reference values used for sarcopenia definition.

Author Country	Sarcopenia Diagnosis Criteria	Muscle Mass Imaging Method Threshold Values for Low Muscle Mass	Muscle Strength Measurement Method Threshold Values for Low Muscle Strength
Jarmusch et al., 2021 [17] Germany	EWGSOP	BIA SMI < 7 kg/m^2^ in men and <5.5 kg/m^2^ in women	Handgrip strength < 27 kg for men and <16 kg for women
Yoo et al., 2021 [18] Republic of Korea	AWGS	DEXA SMI < 7.0 kg/m^2^ in men < 5.4 kg/m^2^ in women	Handgrip Strength < 28 kg in men and <18 kg in women
Bermejo-Bescós et al., 2020 [19] Spain	EWGSOP	BIA SMI 8.51–10.75 and ≤8.50 kg/m^2^ moderate and severe low muscle mass in men; 5.76–6.75 and ≤5.75 kg/m^2^ moderate and severe low muscle mass in women	Handgrip strength < 27 kg for men and <16 kg for women
de Sire et al., 2020 [20] Italy	EWGSOP	BIA SMI < 7.0 kg/m^2^ in men < 5.4 kg/m^2^ in women	Handgrip strength < 27 kg for men and <16 kg for women
Yee et al., 2020 [21] Australia	EWGSOP	BIA SMI adjusted for height; <6.75 kg/m^2^ (female-only study)	Handgrip strength < 16 kg (female only study)
Sánchez-Castellano et al., 2020 [22] Spain	EWGSOP	BIA SMI 8.51–10.75 and ≤8.50 kg/m^2^ moderate and severe low muscle mass in men; 5.76–6.75 and ≤5.75 kg/m^2^ moderate and severe low muscle mass in women	Handgrip strength < 27 kg for men and <16 kg for women
Marzetti et al., 2014 [23] Italy	EWGSOP	BIA SMI < 8.87 kg/m^2^ in men and 6.42 kg/m^2^ in women	Handgrip strength < 30 kg for men and <20 kg for women

**Table 3:** Descriptive summary of sarcopenia biomarkers.

Author Country	Biomarker Class	Biomarker Description
Jarmusch et al., 2021 [17] Germany	Protein	IGF-I and its binding partners were significantly associated with sarcopenia (ß = − 0.360; *p* = 0.047).
Yoo et al., 2021 [18] Republic of Korea	Vitamin	25(OH)D levels were significantly decreased in the sarcopenia group compared with the non-sarcopenia group (*p* = 0.030).
Bermejo-Bescós et al., 2020 [19] Spain	Cytokine	Peripheral levels of IL-6 were significantly higher in the group of participants who died in the year following a hip fracture (*p* = 0.026). Baseline sarcopenia was not associated with mortality (*p* = 0.694).
de Sire et al., 2020 [20] Italy	Protein	Serum myostatin levels significantly decreased in sarcopenic patients receiving amino acid supplementation (*p* = 0.04).
Yee et al., 2020 [21] Australia	Protein	The proportion of sarcopenic individuals detected in the hip fracture group was 60%. Women with hip fractures, compared to the controls, had lower levels of serum albumin (*p* < 0.001), serum insulin-like growth factor-1 (IGF-1) (*p* < 0.001), insulin-like growth factor binding protein 3 (IGFBP-3) (*p* < 0.001), and free testosterone (*p* = 0.001), and impaired beta cell function (*p* = 0.038).
Sánchez-Castellano et al., 2020 [22] Spain	Cytokine	Tumor necrosis factor-α expression was lower in sarcopenic patients than in non-sarcopenic participants (7.9 ± 6.2 vs. 8.3 ± 5.8 pg/mL, *p* < 0.05).
Marzetti et al., 2014 [23] Italy	Peptide	Serum CAF levels were significantly higher in sarcopenic patients relative to non-sarcopenic patients (172.2 ± 47.5 vs. 93.1 ± 44.0 ng/mL, *p* < 0.001).

**Table 4:** BIOCROSS biomarker study quality assessment of the included papers.

Author Country	Hypothesis Objective	Study Population Selection	Study Population Representativeness	Study Population Characteristics	Statistical Analysis	Interpretation and Evaluation of Results	Study Limitations	Specimen Characteristics and Assay Methods	Laboratory Measurement	Biomarker Data Modeling	Total Score
Jarmusch et al., 2021 [17] Germany	2/2	2/2	1/2	1/2	1/2	2/2	2/2	1/2	1/2	1/2	14/20
Yoo et al., 2021 [18] Republic of Korea	2/2	1/2	2/2	1/2	1/2	2/2	1/2	1/2	1/2	1/2	13/20
Bermejo-Bescós et al., 2020 [19] Spain	2/2	2/2	0/2	1/2	1/2	2/2	1/2	1/2	1/2	1/2	14/20
de Sire et al., 2020 [20] Italy	2/2	2/2	1/2	1/2	1/2	2/2	1/2	1/2	1/2	1/2	13/20
Yee et al., 2020 [21] Australia	2/2	2/2	2/2	2/2	2/2	2/2	1/2	0/2	0/2	1/2	14/20
Sánchez-Castellano et al., 2020 [22] Spain	2/2	2/2	1/2	1/2	1/2	2/2	1/2	1/2	1/2	1/2	13/20
Marzetti et al., 2014 [23] Italy	2/2	2/2	2/2	1/2	1/2	2/2	2/2	1/2	1/2	1/2	14/20
